# Maternal Outcomes in Women with Peripartum Cardiomyopathy versus Age and Race-Matched Peers in an Urban US Community

**DOI:** 10.3390/jcdd9080250

**Published:** 2022-08-06

**Authors:** Diana S. Wolfe, Christina Liu, Jack Alboucai, Ariel Karten, Juliet Mushi, Shira Yellin, Julia L. Berkowitz, Shayna Vega, Nicole Felix, Wasla Liaqat, Rohan Kankaria, Thammatat Vorawandthanachai, Anna E. Bortnick

**Affiliations:** 1 Department of Obstetrics and Gynecology, Montefiore Medical Center, Albert Einstein College of Medicine, Bronx, NY 10461, USA; 2 Maternal Fetal Medicine-Cardiology Joint Program, Montefiore Medical Center, Albert Einstein College of Medicine, Bronx, NY 10461, USA; 3 Department of Emergency Medicine, Woodhull Medical and Mental Health Center, Brooklyn, NY 11206, USA; 4 Department of Medicine, North Shore University Hospital, Manhasset, NY 11030, USA; 5 Department of Medicine, Division of Cardiology, New York University Long Island School of Medicine, Mineola, NY 11501, USA; 6 Department of Obstetrics, Gynecology & Reproductive Sciences, Yale University Maternal Fetal Medicine, Greenwich Hospital, Greenwich, CT 06830, USA; 7 Department of Medicine, Mount Sinai Health System, New York, NY 10029, USA; 8 Department of Medicine, Brown University Warren Alpert Medical School, Providence, RI 02903, USA; 9 Department of Obstetrics and Gynecology, Kaiser Permanente Oakland Medical Center, Oakland, CA 94611, USA; 10 Department of Psychiatry and Human Behavior, Brown University Warren Alpert Medical School, Providence, RI 02903, USA; 11 Department of Medicine, New York Health and Hospitals Jacobi Medical Center, Bronx, NY 10461, USA; 12 Department of Medicine, Division of Cardiology, Albert Einstein College of Medicine, Bronx, NY 10461, USA; 13 Department of Obstetrics and Gynecology, State University of New York Downstate, Brooklyn, NY 11203, USA; 14 Department of Medicine, Division of Geriatrics, Montefiore Medical Center, Albert Einstein College of Medicine, Bronx, NY 10461, USA

**Keywords:** cardiomyopathy, pregnancy, heart failure

## Abstract

Peripartum cardiomyopathy (PPCM) is idiopathic systolic congestive heart failure around pregnancy. Comparisons with matched controls are lacking. We investigated maternal characteristics and outcomes up to 12 months in a cohort admitted to Montefiore Health System in Bronx, New York 1999–2015 (*n* = 53 cases and *n* = 92 age and race-matched controls, >80% Black or Hispanic/Latina). Compared to peers, women with PPCM had more chronic hypertension (24.5% vs. 8.8%, *p* = 0.001), prior gestational hypertension (20.8% vs. 5.4%, *p* = 0.001), prior preeclampsia (17.0% vs. 3.3%, *p* = 0.001), familial dilated cardiomyopathy (5.7% vs. 0.0%, *p* = 0.04), smoking (15.1% vs. 2.2%, *p* = 0.001), lower summary socioeconomic scores (−4.12 (IQR −6.81, −2.13) vs. −1.62 (IQR −4.20, −0.74), *p* < 0.001), public insurance (67.9% vs. 29.3% *p* = 0.001), and frequent depressive symptoms. Women with PPCM were often admitted antepartum (34.0% vs. 18.5%, *p* = 0.001) and underwent Cesarean section (65.4% vs. 30.4%, *p* = 0.001), but had less preterm labor (27.3% vs. 51.1%, *p* = 0.001). Women were rarely treated with bromocriptine (3.8%), frequently underwent left ventricular assist device placement (9.4% and *n* = 2 with menorrhagia requiring transfusion and progesterone) or heart transplantation (3.8%), but there were no in-hospital deaths. In sum, women with PPCM had worse socioeconomic disadvantage and baseline health than matched peers. Programs addressing social determinants of health may be important for women at high risk of PPCM.

## 1. Introduction

Maternal mortality is rising in the US, driven by cardiovascular disease among Black and Native women [1]. Peripartum mortality in Black women is 2.5-fold higher than non-Hispanic White women nationally [2]. Peripartum cardiomyopathy (PPCM) is an important cause, a rare form of idiopathic acute left systolic congestive heart with unclear etiology. PPCM affects approximately 0.05–0.1% of women with live births [3]. PPCM is defined as idiopathic cardiomyopathy in which the patient has a left ventricular ejection fraction (LVEF) ≤ 45% in the last month of pregnancy or up to 5 months postpartum [4]. Women of African descent are at greater risk for developing PPCM compared to White women and the prevalence of PPCM in Black women is >5-fold higher than in non-Black women in the US [5]. Black women with PPCM were found to have significantly longer-lasting heart dysfunction, need for heart transplant, and death, as compared to White women with PPCM [6]. Additional risk factors include preeclampsia, eclampsia, diabetes mellitus, smoking, hypertension, multi-gestational pregnancy, and older maternal age [7]. A retrospective analysis of pregnancy-associated deaths in the state of California found that the majority were due to PPCM and that >60% were possibly preventable with early recognition and management [8]. There may be a genetic predisposition to PPCM in women with a common TTN truncating variant gene, but this appears to be relevant only in some [9].

The prognosis of PPCM is highly variable as most women recover left ventricular function [10]. Others progress to end-stage dilated cardiomyopathy and require advanced heart failure therapy in the form of left ventricular assist devices (LVAD) or heart transplantation. Furthermore, abnormal echocardiogram characteristics have been reported among those PPCM patients presumed to be recovered, either due to residual global longitudinal strain or persistent arterial stiffness [11,12].

Even when LVEF recovers, there is an elevated risk for recurrent PPCM in future pregnancies. Elkayam et al. reported a study of subsequent pregnancies in two groups, those with and without fully recovered LVEF. Recurrence of PPCM was 20% in those with recovered LVEF versus 50% in those who did not recover prior to a repeat pregnancy [13]. 

Cohort studies of PPCM are limited by small numbers due to the rarity of the disease and case–control comparisons have been mostly limited to Black vs. White women, with a lack of information about other race/ethnic groups. The purpose of our study was to investigate maternal outcomes in age and race-matched women with PPCM from a large urban minority community burdened by high maternal mortality, to improve understanding of the modifiable risk factors and health disparities that could play a role in this disease apart from race alone, by characterizing the subgroup who developed the disease and evaluating their 12-month outcomes. We hypothesized that worse socioeconomic status and hypertensive disorders would be more prevalent in women with PPCM and that they would have worse cardiovascular outcomes than age and race-matched peers.

## 2. Materials and Methods

The Montefiore Health System (Bronx, NY, USA) electronic medical record was queried using the Looking Glass Clinical Analytics search engine (LGCA, Streamline Health, Atlanta, GA, USA), for women hospitalized between 1999–2015 with International Statistical Classification of Diseases and Related Health Problems, 9th revision diagnosis and procedure codes (ICD)−9 codes for PPCM or heart failure or current procedural terminology (CPT) for heart transplantation or left ventricular assist device placement (674.53, 674.54, 428.0, 428.1, 428.21, 428.22, 428.23, 428.40, 428.41, 428.43, 428.9, 37.5, 37.51, 37.62, 37.65). Briefly, inclusion criteria for cases were: age ≥16 years, peripartum LVEF ≤ 45%, and idiopathic, non-ischemic cardiomyopathy, diagnosis during pregnancy or up to 5 months after delivery. Exclusion criteria were: missing LVEF, missing documentation relating low LVEF to the peripartum period, and other possible causes of low LVEF such as congenital heart disease, valvular disease, coronary artery disease, septicemia, cocaine/alcohol abuse, history of exposure to chemotherapy or chest irradiation, eclampsia, autoimmune disease, supraventricular arrhythmia and infection with human immunodeficiency virus. Controls were randomly selected (1.7 controls/case) from all pregnant women presenting between 2003–2015 to reflect similar care patterns as cases and were individually matched to cases by year of birth, as well as self-reported race/ethnicity. The study was approved by the Institutional Review Board of the Albert Einstein College of Medicine.

### Demographic and Clinical Variables

Charts were analyzed for baseline characteristics, and medical and obstetric history. Demographics were obtained using LGCA, clinical outcomes were obtained from the EMR by trained abstractors. Age was defined as interval from date of birth to date of delivery or if not available for PPCM cases, date of diagnosis with PPCM. Race/ethnicity, country of origin, and preferred language was self-report. Summary socioeconomic score (SSS) was defined as previously described [14]. Briefly, 6 variables comprise a composite score representing dimensions of education, occupation, wealth, and income and expressed as a standard deviation from the mean of the census block group, a proxy for neighborhood. Lower SSS indicates worse socioeconomic status as compared to the neighborhood. Gravidity and parity were self-reported. Body mass index (BMI) was calculated as weight (kg) divided by height squared (m^2^) and obesity was defined as BMI ≥30. Chronic hypertension (HTN) was defined by history or treatment with antihypertensive medication. Gestational hypertension was defined by treatment with antihypertensive medication only in pregnancy. Pre-gestational diabetes was defined by history or past use of anti-diabetic medication. Gestational diabetes was defined as diabetes diagnosis or use of anti-diabetic medication only in pregnancy. Smoking was defined as any cigarette use in pregnancy by self-report. Any alcohol and other substance use in pregnancy were defined by self-report. Prior history of PPCM was by history or by chart review from a trained abstractor using the above inclusion and exclusion criteria. Psychiatric history was defined as documented diagnosis of generalized anxiety disorder, major depressive disorder, bipolar I or II disorder, or schizophrenia. Anxiety or depressive symptoms were recorded as documented, and psychiatric medications and timing of prescription relative to the pregnancy were noted. End-stage renal disease was defined as a history of dialysis. Asthma was defined by history or use of a bronchodilator or long-acting inhaled steroid. Insurance status was documented on admission to the hospital. LVEF was obtained from baseline transthoracic echocardiogram (TTE) and repeat TTE closest to 12 months of follow-up.

Obstetrics outcome measures were, preeclampsia; systolic blood pressure (SBP) ≥ 140 mm Hg or diastolic blood pressure (DBP) ≥ 90 mm Hg, on 2 occasions, at least 4 h apart, in a previously normotensive woman, and protein: creatinine ≥0.3 or dipstick reading = 1+, or severe features of SBP ≥160 mm Hg or DBP ≥110 mm Hg, on 2 occasions at least 4 h apart, thrombocytopenia, elevated liver function tests twice normal, serum creatinine >1.1 mg/dL or doubling of serum creatinine, visual or cerebral symptoms, and eclampsia was additionally defined by seizure. Pregnancy-induced hypertension was defined as SBP >140 mmHg and DBP >90 mmHg. Gestational diabetes was defined as abnormal serum glucose levels following a 100 mg oral glucose load. Chronic abruption was defined as delay of labor at least 7 days after initial hemorrhage. Placenta previa was defined as the placenta over the opening of the cervix. Preterm labor was defined as dilation of the cervix after week 20 and before week 37 of pregnancy and premature prolonged rupture of membranes was defined as rupture of the amniotic sac before the onset of labor. Ectopic pregnancy was defined as pregnancy outside of the uterus. Postpartum hemorrhage was defined as uterine bleeding leading to blood transfusion. Chorioamnionitis was defined as placental infection. Placenta accreta was defined as invasion of the placenta through the uterus, and labor dystocia as prolonged delivery. Rehospitalization was defined as any hospital admission lasting ≥24 h in the Montefiore Health System within 12 months after delivery or diagnosis of PPCM. Study data were collected in REDCap and analyzed in STATA 15 (College Station, TX, USA). Baseline characteristics and outcomes were expressed as mean standard deviation for normally distributed continuous data, median (interquartile range) for skewed data, or frequency and proportion for categorical data. We used Student’s t-test for normally distributed continuous data, Mann–Whitney for skewed continuous data, Pearson’s chi-square for categorical data for >5 observations, and Fisher’s exact for categorical data with <5 observations. Statistical significance was two-tailed *p* < 0.05.

## 3. Results

**Figure 1:**
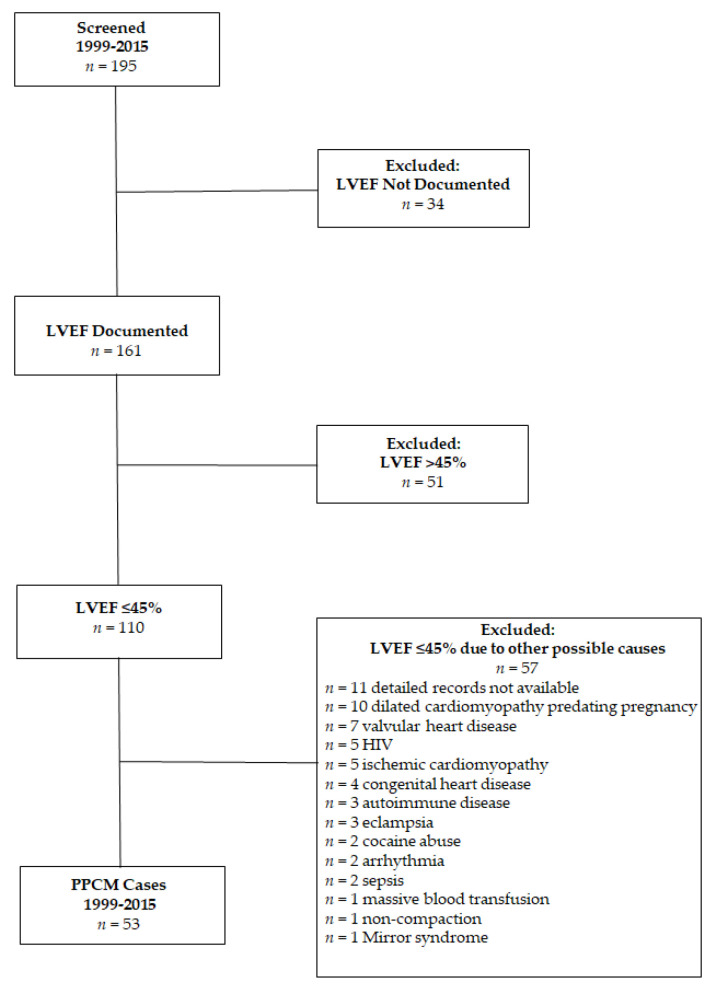
Flowchart of inclusion and exclusion of peripartum cardiomyopathy cases. HIV = human immunodeficiency virus; LVEF = left ventricular ejection fraction; Mirror syndrome = preeclampsia, fetal hydrops, and parvovirus infection; PPCM = peripartum cardiomyopathy.

Of the 195 potential PPCM cases identified, 53 met the inclusion criteria (Figure 1). The majority were excluded due to an LVEF > 45%, no LVEF information, or alternative diagnoses potentially causing or contributing to left systolic congestive heart failure. Women with PPCM were well-matched by age (32 ± 7 vs. 30 ± 7 years, *p* = 0.96) and race/ethnicity (88.7% vs. 84.7% Black or Hispanic/Latina, *p* = 0.58) with 92 peer controls (Table 1). The cohort was primarily US-born and English speaking. The neighborhood SSS z score was statistically significantly lower in women with PPCM than in controls (−4.12 (IQR −6.81, −2.13) vs. −1.62 (IQR −4.20, −0.74), *p* < 0.001). Women with PPCM and controls had similar gravidity and parity, as well as prevalent obesity. Chronic hypertension, prior gestational hypertension, prior preeclampsia, prior history of PPCM, family history of dilated cardiomyopathy, ESRD, smoking, and alcohol use in pregnancy were higher in women with PPCM than in controls. Women with PPCM were also more likely to be publicly insured.

Women in the community had a high psychiatric burden overall. The prevalence of prior psychiatric history, formal generalized anxiety, major depressive disorder, bipolar disorder, and schizophrenia diagnoses was comparable between the groups and anxiety symptoms were not significantly different amongst cases and controls (Table 2). Both groups were likely to be treated with therapy or medication postpartum and there were no differences in medication class used to treat either group. However, depressive symptoms were more prevalent in women with PPCM, and they were more often treated with medication in the preconception period than controls. The diagnosis of depressive symptoms was frequently diagnosed postpartum in women with PPCM.

In the hospital, 86.8% of patients with PPCM were treated with beta-blockers, 81.1% with diuretics, and 73.6% with angiotensin-converting enzyme inhibitors or angiotensin receptor blockers while the use of bromocriptine was only 3.8% (Table 3). Women with PPCM had a severely reduced LVEF at diagnosis of 29 ± 11% (Table 4). There was a significant improvement in LVEF over a median follow-up of 1.27 years for the 34 patients who had available repeat TTE, to 43 ± 14%. Women with PPCM were frequently critically ill and rehospitalized with acute CHF exacerbations in the following 12 months (Table 4). The majority were seen by an advanced heart failure specialist. While a high proportion received left ventricular assist devices (LVADs) and underwent heart transplantation, there were no in-hospital deaths. Two women receiving LVADs developed major vaginal bleeding requiring blood transfusion and acute treatment with progesterone.

With respect to obstetric outcomes, women with PPCM had 55 intrauterine pregnancies and controls had 97, with a comparable frequency of live births (92.3 % vs. 97.8%, *p* = 0.19) and twin gestations (3.9% vs. 5.4%, *p* = 0.27), respectively. Women with PPCM were more frequently admitted during pregnancy than controls (34.0% vs. 18.5%, *p* = 0.001, Table 5). Women with PPCM and controls had a similar frequency of hypertensive disorders and gestational diabetes but were less likely to have preterm labor or preterm premature rupture of membranes (PPROM) than controls (27.3% vs. 51.1%, *p* = 0.001). Women with PPCM were more often delivered via Cesarean (C) section (65.4% vs. 30.4%, *p* = 0.001) and most were primary, though the frequency of primary C-section did not differ by group (53.3% vs. 50.0%, *p* = 0.80). The indication for C-section was often a maternal cardiovascular cause or preeclampsia and this was comparable between groups (34.5% vs. 30.8%, *p* = 0.77). There were no statistically significant differences in other intrapartum maternal outcomes. Readmission for thrombotic, bleeding, and infectious outcomes were comparable between women with PPCM and in controls up to 12 months of follow-up (Table 5).

## 4. Discussion

We studied women with an ICD-9 or -10 diagnosis of PPCM between 1999–2015 from a single academic center serving a large, minority urban community, comparing their demographic, clinical characteristics, and outcomes with contemporary age and race-matched controls. Women were often miscoded as having PPCM due to other diagnoses leading to pulmonary edema or acute left systolic congestive heart failure. This has implications for PPCM studies using ICD-9 or -10 codes where medical charts cannot be audited for accuracy. Cases were markedly more impoverished than peers and often publicly insured. Both cases and controls had a similar burden of major co-morbidities such as diabetes and obesity, though women with PPCM had a higher burden of hypertension or preeclampsia and more often had adverse health behaviors of smoking or alcohol use in pregnancy. In this cohort, women with PPCM had more frequent antepartum admission and C-section, but less pre-term labor.

The higher burden of hypertension or preeclampsia in women with PPCM is consistent with the current literature reporting a strong association between preeclampsia and PPCM [15]. PPCM shares some similarities in pathophysiology with preeclampsia, a hypertensive disorder due to placental factors [10]. Preeclampsia occurs in 7% of all pregnant patients and can persist into the postpartum period [16]. Preeclampsia is a placental disease as there is direct contact between the maternal immune system and the semi-allogeneic trophoblast [17]. Syncytiotrophoblast stress results in an imbalanced ratio of the soluble fms-like tyrosine kinase 1 (sFLT-1), a circulating anti-angiogenic factor that binds and antagonizes the placental growth factor. Animal studies have shown that persistent sFLT-1 results in PPCM, which may be a mechanistic link between preeclampsia and PPCM [18]. 

Women with PPCM in this study were also more likely to have had a recurrence or a family history of dilated cardiomyopathy, consistent with previous studies which suggest that a subset of patients with PPCM may be hereditary [19]. Genetic screening was not available in this cohort and thus, the true frequency of familial dilated cardiomyopathy may be underestimated. Women with this family history may benefit from closer monitoring in pregnancy.

There was a high burden of psychiatric diagnoses and symptoms amongst cases and controls, though women with PPCM had more depressive symptoms and were more likely to have received medical treatment preconception than controls, a finding consistent with a prior report of mood disorders and depression, in particular, as a predictive risk factor for PPCM [20]. Depression, combined with low material resources, may contribute to poor self-care, substance use, appetite dysregulation, and heightened stress responses [21]. Higher neighborhood disadvantage is associated with cardiac dysfunction [22]. Mechanisms relating social determinants of health to worse cardiometabolic outcomes include racism in healthcare and education, higher social and financial stress, poor neighborhood conditions, and/or more negative life events [2,22].

Anxiety has also been posited as a risk factor associated with PPCM in a study of Black vs. White patients; however, in our study, anxiety was comparably prevalent in cases and controls [20]. There is growing evidence of mental health disorders among PPCM patients, with depression as high as 40–65% in some studies [23]. Thus, mental health support should be anticipated for women with PPCM. This may be especially important for those with LVAD or heart transplantation who experience increased medical complexity. In sum, genetic predisposition, in combination with hypertension, preeclampsia, socioeconomic and psychosocial stress, may drive PPCM.

In this cohort, the average LVEF significantly improved from diagnosis of PPCM to 12 months, but only 23.5% of patients recovered to an LVEF >55%, which is lower than the 61% recovery rate reported in the literature [24]. This finding could be related to worse health status and variability in the medical treatment of PPCM. A large number underwent LVAD implant or heart transplantation. Notably, two women with LVAD developed major vaginal bleeding, possibly due to the combination of anticoagulation and acquired von Willebrand deficiency, which has been observed with continuous flow pumps [25]. This observation, in addition to reports of pregnancies carried out by women with LVADs, emphasizes the importance of gynecologic and contraceptive care in this group [26]. Consultation with obstetrics and gynecologists should be part of the routine LVAD evaluation for individuals who could become pregnant. Long-acting reversible contraceptives (LARC) are safe in this patient population. Progesterone-secreting intrauterine devices (IUDs) and subcutaneous implants thin the uterine lining, decreasing menses, which could be favorable to prevent anticoagulation-associated hemorrhage [27].

Treatment patterns in this study indicated a preference for beta-blockers, angiotensin-converting enzyme inhibitors and angiotensin receptor blockers, hydralazine, isosorbide, and aldosterone receptor antagonists with less use of anticoagulation and bromocriptine during this time period. We did not observe a higher number of thrombotic events in women with PPCM as compared to controls, but the incidence of LV thrombus is rare in PPCM, roughly 1% in the National Inpatient Sample; thus, the study was underpowered for this outcome [28]. Alternatively, there may have been fewer women in this cohort with connective tissue disorders or preexisting coagulopathy.

There is emerging data suggesting that bromocriptine could be a beneficial therapy for LV recovery. A randomized controlled trial of 63 patients found that bromocriptine treatment was associated with higher rates of recovery and lower rates of morbidity in women with PPCM, with full recovery in 52% of those treated for 7 days and 68% among those treated for 8 weeks, but the study lacked a placebo arm [29]. Another randomized study of 30 patients found that only 37% of their cohort treated with bromocriptine fully recovered [30]. Both studies were limited by a small sample size. Large randomized controlled trials are needed to establish safety and efficacy to reconcile conflicting findings. The REBIRTH trial (www.clinicaltrials.gov NCT05180773) is pending enrollment to address whether bromocriptine should be a standard of care for women with PPCM.

The strengths of this study are several. We gathered detailed information on a large number of women with PPCM living in a diverse, multi-ethnic, urban US community. A significant number of women received advanced heart failure therapy in this cohort. However, the study was limited to retrospective data, outcomes were collected from readmission to the same health system and included women presenting to a quaternary care academic center which may not be generalizable to different sites. As some PPCM women delivered at outside centers, we did not have complete data for all neonates and did not compare fetal outcomes.

Preventing, screening for and treating PPCM is a significant priority to reduce morbidity and mortality for women around the time of pregnancy. Defining optimal medical therapy and delivery of comprehensive social and mental health support are aspects for future research to decrease adverse events in women with PPCM.

## 5. Conclusions

In conclusion, this study expands our understanding of demographic and clinical characteristics in addition to treatment patterns of women with PPCM in a US urban race/ethnic minority community. It further supports that apart from age and race/ethnicity, women with PPCM were more likely to experience abject poverty, smoking or alcohol use in pregnancy, hypertension, preeclampsia, prepartum depression, a hereditary tendency to cardiomyopathy, antepartum admission and C-section. Women with a high-risk profile may benefit from careful monitoring throughout pregnancy with connection to social, financial, physician, nursing, doula, midwifery, and/or community health services. There was poor LVEF recovery for many in this cohort and high conversion to advanced heart failure therapies with LVAD which requires significant social support already lacking in this group. Additionally, LVADs were sometimes associated with significant menorrhagia; thus, women should be adjunctly offered IUD placement or progesterone-containing subcutaneous implants for the dual effect of contraception and to thin the endometrium. Treatment of PPCM requires a multidisciplinary approach involving maternal-fetal medicine and cardiology services thus, cardio-obstetrics programs may streamline care. Whether aspirin therapy for the prevention of preeclampsia or bromocriptine to block prolactin impacts the incidence of or recovery from PPCM is unknown. Action is needed to investigate the interaction of social determinants of health, risk factors, novel treatments, and lasting effects of PPCM on cardiovascular health, so as to reduce maternal mortality in Black and Native women across the US.

## Figures and Tables

**Table 1:** Baseline characteristics of women with peripartum cardiomyopathy (*n* = 53) vs. age and race-matched peers (*n* = 92) in the Montefiore Peripartum Cardiomyopathy Cohort.

Covariate	PPCM (*n* = 53)	Controls (*n* = 92)	*p* Value
Age, y	32 ± 7	30 ± 7	0.96
Race *n*, %			0.58
Black/African American	32, 60.4	51, 55.4	
Hispanic/Latina	15, 28.3	27, 29.3	
Non-Hispanic White	2, 3.8	6, 6.5	
Other	2, 3.8	5, 5.4	
Southeast Asian	1, 1.9	2, 2.2	
Not reported	1, 1.9	1, 1.1	
Black/African American	32, 60.4	51, 55.4	
Country of origin outside US	4, 7.6%	11, 12.0%	0.57
English as preferred language	46, 86.8	87, 94.6	0.10
Summary Socioeconomic Score	−4.12 (−6.81, −2.13)	−1.62 (−4.20, −0.74)	<0.001
Gravida *n*	3 (2, 5)	3 (2, 5)	0.70
Para *n*	2 (1, 3)	1 (0, 2)	0.16
Body mass index ≥30 *n*, %	30, 60.0	28, 43.1	0.07
Chronic hypertension *n*, %	13, 24.5	8, 8.8	0.001
Prior gestational hypertension *n*, %	11, 20.8	5, 5.4	0.001
Prior preeclampsia *n*, %	9, 17.0	3, 3.3	0.001
Asthma *n*, %	9, 17.0	18, 19.6	0.70
Prior history of PPCM *n*, %	7, 13.2	0, 0.0	0.001
Family history of dilated cardiomyopathy *n*, %	3, 5.7	0, 0.0	0.04
Chronic diabetes *n*, %	3, 5.7	2, 2.2	0.27
Lupus or rheumatoid arthritis *n*,%	0, 0.0	3, 3.3	0.18
Hypothyroidism *n*, %	2, 3.8	3, 3.3	0.87
Hyperthyroidism *n*,%	1, 1.9	1, 1.0	0.69
End-stage renal disease *n*, %	1, 1.9	0, 0.0	0.001
Smoking during pregnancy *n*, %	8, 15.1	2, 2.2	0.001
Alcohol during pregnancy *n*, %	2, 3.8	0,0.0	0.001
Insurance *n*, %			0.001
Public	36, 67.9	27, 29.3	
Commercial	13, 24.5	40, 43.5	
Uninsured	2, 3.8	22, 23.9	
Not reported	2, 3.8	3, 3.3	

Values are mean ± standard deviation or median (interquartile range). PPCM = peripartum cardiomyopathy.

**Table 2:** Psychiatric history of women with PPCM (*n* = 53) vs. age and race-matched controls (*n* = 92).

Covariate	PPCM (*n* = 53)	Controls (*n* = 92)	*p* Value
Prior psychiatric history *n*, %	16, 30.2	20, 21.7	0.26
Any active psychiatric diagnosis *n*, %	15, 28.3	14, 15.2	0.06
Depressive symptoms	7, 13.2	0, 0.0	0.001
Depressive symptoms diagnosed postpartum	9, 17.0	3, 3.3	0.009
Depressive symptoms treated with medication	9, 17.0	4, 4.4	0.02
Treated with psychiatric medication prepartum	3, 5.7	0, 0.0	0.047
Treated with psychiatric medication postpartum	4, 7.6	3, 3.3	0.26
Anxiety symptoms	8, 15.1	5, 5.4	0.05

**Table 3:** In-hospital cardiac medications for women diagnosed with PPCM (*n* = 53).

In-Hospital Cardiac Medications *n*, %	
Beta blocker	46, 86.8
Diuretic	43, 81.1
ACEi or ARB	39, 73.6
Warfarin	11, 20.8
Aldosterone receptor antagonist	12, 22.6
Hydralazine and/or isosorbide mononitrate or dinitrate	11, 20.8
Digoxin	11, 20.8
Aspirin	7, 13.2
Inotrope	5, 9.4
Nitroglycerin	3, 5.7
Calcium channel blocker	3, 5.7
Bromocriptine	2, 3.8
Methyldopa	1, 1.9

ACE = Angiotensin-converting enzyme inhibitor; ARB = angiotensin receptor blocker.

**Table 4:** Cardiac outcomes in women with PPCM (*n* = 53).

Cardiac Outcomes		*p* Value
LVEF at diagnosis, % LVEF at 12-month follow-up (*n* = 34) *, %	29 ± 11 43 ± 14	<0.0001
Follow-up with CHF subspecialist *n*, %	28, 52.8	
12-month CHF rehospitalization, *n* %	13, 24.5	
ICD *n*, %	9, 16.9	
Mechanical ventilation *n*, %	7, 13.2	
LVAD, *n*, %	5, 9.4	
Heart transplant, *n*, %	2, 3.8	
Intra-aortic balloon pump	1, 1.9	
In-hospital maternal death *n*, %	0, 0.0	

* Median follow-up 1.27 (0.96, 1.81) years. Values are mean ± standard deviation or median (interquartile range). CHF = congestive heart failure; ICD = intracardiac defibrillator; LVAD = left ventricular assist device; LVEF = left ventricular ejection fraction; PPCM = peripartum cardiomyopathy.

**Table 5:** Antepartum, intrapartum, and non-cardiac 12 month outcomes in women with PPCM (*n* = 53) vs. controls (*n* = 92).

Antepartum Maternal Outcomes	PPCM (*n* = 53)	Controls (*n* = 92)	*p* Value
Antepartum admission *n*, %	18, 34.0	17, 18.5	0.001
Preeclampsia *n*, % Pregnancy induced hypertension *n*, %	10, 18.9 1, 1.9	11, 12.0 0, 0.0	0.08 0.37
Gestational diabetes *n*, %	8, 15.1	7, 7.7	0.12
Chronic abruption *n*, %	1, 1.9	1, 1.1	0.52
Placenta previa *n*, %	0, 0.0	2, 2.2	0.36
Preterm labor, PPROM *n*, %	12, 27.3	47, 51.1	0.001
Intrauterine fetal demise *n*, %	1, 1.9	1, 1.1	0.69
Ectopic pregnancy *n*, %	1, 1.9	0, 0.0	0.19
Termination of pregnancy *n*, %	1, 1.9	0, 0.0	0.19
**Intrapartum maternal outcomes**			
Cesarean section *n*, %	34, 65.4	28, 30.4	0.001
Post-partum hemorrhage *n*, %	5, 9.4	2, 2.2	0.05
Placental abruption *n*, %	2, 3.8	2, 2.2	0.57
Chorioamnionitis *n*, %	1, 1.9	2, 2.2	0.91
Placenta accreta spectrum *n*, %	1, 1.9	1, 1.1	0.69
Eclampsia *n*, %	0, 0	1, 1.1	0.45
Labor dystocia *n*, %	0, 0	1, 1.1	0.45
**Non-cardiac maternal outcomes 12 months postpartum**			
Abscess, sepsis, infection *n*, %	3, 5.7	1, 1.1	0.14
Stroke, DVT, PE *n*, %	2, 3.8	1, 1.1	0.55
Bleeding, incisional *n*, %	0, 0.0	1, 1.1	1.00

DVT = deep vein thrombosis; PE = pulmonary embolism; PPROM = preterm premature rupture of membranes.

## Data Availability

Partial data were presented at the 5th International Conference on Cardiovascular Problems in Pregnancy (CPP 2018), Bologna, Italy.

## Abbreviations

PPCM	Peripartum cardiomyopathy
LVEF	Left ventricular ejection fraction
